# Supplementary material for: Knowledge, Attitudes and Practices of Veterinarians Towards Antimicrobial Resistance and Stewardship in Nigeria
Source: Antibiotics (Basel). 2020 Jul 28;9(8):453. doi: 10.3390/antibiotics9080453 (PMC7460309; doi:10.3390/antibiotics9080453)
Supplement: Supplementary file 1 [file antibiotics-09-00453-s001.zip › Supp Material S1 - Questionnaire.pdf]

Supplementary material S1: Questionnaire

Your participation in this survey is valued. Before proceeding to complete the survey, please indicate below your consent to participate by checking the appropriate option.

- Yes, I consent
- No, I do not consent

Which University did you graduate from? Tick as appropriate.

- Ahmadu Bello University (ABU), Zaria
- University of Ibadan (UI), Ibadan
- University of Nigeria Nsukka (UNN)
- Usmanu Danfodiyo University, Sokoto (UDUS)
- University of Maiduguiri
- University of Abuja
- Federal University of Agriculture, Abeokuta (FUNAAB)
- Federal University of Agriculture Makurdi (FUAM)
- Other, please specify ____

What is the highest level of education you attained?

- Veterinary degree (DVM, etc)
- MSc/MPH
- Fellow, College of Veterinary Surgeon (FCVS)
- PhD
- Others, please specify ____

For how many years have you been registered as a veterinary surgeon? Tick as appropriate.

- 0-<5 years
- 5-10 years
- 10-15 years
- 15-20 years
- 21 and above
- Prefer not to say

What is your gender?

- Male
- Female
- Prefer not to say

Please indicate your age group: Tick as appropriate

- 18-24 years old
- 25-34 years old
- 35-44 years old
- 45-54 years old
- 55-64 years old
- 65 years or older
- Prefer not to say

Which of the following best describes your employment status? Tick as appropriate.

- Private practice
- Non-governmental organisation employee
- Government employee
- Research
- Teaching
- Other, please specify ____

What type of veterinary practice do you work in? Tick all that apply.

- Large animal practice (cattle, horse, goat, sheep, pig)
- Small animal practice (dogs, cats, rabbits)
- Poultry practice (chicken, turkey)
- Exotics practice (parrots, tortoise, snakes, etc)
- Wildlife practice (wild animals)
- Fish practice
- Mixed practice (large, small or exotic animals)
- Do not practice
- Other, please specify ____

In what state of Nigeria is your practice located?

In your opinion, which of the following are important challenges in the world today? [32] Select all that apply.

- Climate change
- Food security
- Antibiotic resistance
- Gender inequality
- Obesity

In your opinion, please indicate if the following statements are true or false. [32]

	True	False	Unknown
Antibiotics kill or stop the growth of both good and bad bacteria			
Antibiotics kill viruses			
A withdrawal period has to be strictly observed in treated poultry before any poultry product is passed as fit for human consumption			
Overuse of antibiotics makes them become ineffective			
Bacteria can become resistant to antibiotics			
A withdrawal period does not have to be observed for milking cows treated with antibiotics such as penicillin before milk can be consumed			

What is the difference between an antimicrobial and an antibiotic? [32] Select one.

- Antibiotics refers only to naturally occurring compounds, it does not include synthetic compounds
- An antibiotic is the same as an antimicrobial
- Antibiotics refer to drugs that kill and or stop the growth of bacteria, whereas antimicrobials include drugs that kill viruses, fungi or bacteria
- Don't know

Have you ever heard of the term ‘antibiotic stewardship’ or ‘antimicrobial stewardship’? [32]

- Yes
- No

If you answered yes to the previous question, which of the following do you think best defines antibiotic stewardship or antimicrobial stewardship?

- A systematic effort to educate and persuade prescribers of antibiotics to follow evidence-based prescribing, in order to stem antibiotic overuse, and thus antibiotic resistance.
- A coordinated intervention designed to improve and measure the appropriate use of antibiotics by promoting the selection of the optimal antimicrobial drug regimen, dose, duration of therapy and route of administration.
- Optimal selection, dosage, and duration of antibiotic treatment that results in the best clinical outcome for the treatment or prevention of infection, with minimal toxicity to the patient and minimal impact on subsequent resistance
- All of the above
- None of the above

Have you read or heard of the Nigeria Center for Disease Control 5-point action plan for responsible use of antimicrobials?

- Yes
- No

Of the following, which topics would you like to receive more information on? [32] Select all that apply.

- Guidelines to use of antibiotics
- Resistance to antibiotics and how resistance develops
- Animal disease conditions for which antibiotics are used or should be used
- Links between the health of humans, animals and the environment
- Microbiological culture and sensitivity testing
- Critically important antimicrobials

Of the following potential contributors to antibiotic resistance, which ones do you consider the most important? [32] Select all that apply.

- Long duration of antibiotic treatments
- Under-dosing of antibiotics
- Excessive use of antibiotics in livestock and food production
- Poor biosecurity practices
- Too many broad-spectrum antibiotics used
- Public awareness of antibiotic resistance

To what extent do you agree or disagree with the following statements? [32]

	Disagree	Neither agree/nor disagree	Agree
Healthy people and animals can carry antibiotic resistant bacteria			
Biosecurity is important in food production			
When there is poor biosecurity, prophylactic antibiotics are an appropriate alternative to protect animal health			

Please answer each of the following questions: [32]

	Yes	No	Unknown
Do you believe that prescribing unnecessary antibiotics is professionally unethical?			
Do you think we have enough antibiotics under development at the moment to keep up with the problem of resistance?			
Do you think that antibiotic resistance is a national problem?			
Do you think that antibiotic resistance will be a greater problem later in your career than it is today?			
Do you believe that the antibiotics you prescribe may contribute to the problem of antibiotic resistance?			
Do you feel you have sufficient knowledge on antibiotic use?			

In what microbes is antibiotic resistance a major problem? [32] Select all that apply.

- Bacteria
- Fungi
- Viruses
- Protozoa
- Helminths (gastrointestinal worms)

Do you think overuse of antibiotics can lead to development of resistant organisms?

- Yes
- No
- Don’t know

Do you believe as a veterinarian, you have a role to play in preventing public health threats posed by antibiotic resistance?

- Yes
- No
- Don’t know

Does your practice have a standardised protocol for treating sick animals with antibiotics?

- Yes
- No
- Don't know

How often do you encounter owner-initiated antibiotic treatment before presentation?

- Frequently (> 3 times a month)
- Sometimes (1-3 times a month)
- Never
- Don't know

When a patient presents for the first time and antibiotics are indicated, what guidelines do you follow to help you select the appropriate antibiotic? Select all that apply.

- Microbiological culture and sensitivity (MCS) testing
- Empirical treatment whilst awaiting MCS result
- Use whatever antibiotic is available
- Use whatever antibiotic the patient can afford
- Don't know

How often do you undertake antimicrobial sensitivity testing before starting antibiotic treatment?

- Never
- Sometimes (1-3 times a month)
- Frequently (more than 3 times a month)
- Don't know

Which of the following influence your decision the most important when requesting antimicrobial sensitivity testing? [45] Select all that apply.

- Poor response to initial antimicrobial therapy or therapeutic failure
- Recurrent conditions
- Owner request
- No knowledge of farm or animal health status

Of the following barriers to using antimicrobial sensitivity testing, which barriers do you consider the most important? Select all that apply.

- Unavailability of lab services to conduct microbiological culture and sensitivity testing
- Owner unable or not willing to pay for culture and sensitivity testing
- Case is urgent, requires immediate antibiotic therapy
- Not sure of what to request from the lab
- Long waiting time for sensitivity results

Which of the following guidelines influence your decision the most when selecting an antibiotic to use? Select all that apply.

- Prescription guidelines/policies supplied by vet hospital/bodies/entities (literature/brochures)
- Legal restriction of drug to a defined species
- Product label/leaflet (manufacturer recommendations)
- Published literature
- Veterinary education/training

Which of the following factors influence your decision the most important when selecting an antibiotic to use? Select all that apply.

- Cost of antibiotic drug
- Marketing offers
- Drug sellers
- Advertisement by drug representative
- Owner ability to pay
- Profit margin to vet

Which of the following past experiences influence your decision the most important when selecting an antibiotic to use? Select all that apply.

- Owner preference for a specific antibiotic
- Vet previous experience
- Advice from colleagues

Of the following antibiotic characteristics, which ones influence your decision the most important when selecting an antibiotic to use? Select all that apply.

- Withdrawal period
- Sensitivity results/antibiogram
- Risk of development of antibiotic resistance
- Spectrum of action of antibiotic
- Ease of administration
