# Supplementary material for: Knowledge, Attitudes and Practices of Veterinarians Towards Antimicrobial Resistance and Stewardship in Nigeria
Source: Antibiotics (Basel). 2020 Jul 28;9(8):453. doi: 10.3390/antibiotics9080453 (PMC7460309; doi:10.3390/antibiotics9080453)
Supplement: Supplementary file 1 [file antibiotics-09-00453-s001.zip › Supp Materials Tables S1-S9.pdf]

Supplementary Materials: Tables S1-S9

Table S1. Knowledge of appropriate antibiotics use and AMR among respondents

Variables	Response	Frequency (n=241)	Percentage (%)
Antibiotics kill or stop the growth of both good and bad bacteria	True	220	91.3
	False	20	8.3
	Unknown	1	0.4
Antibiotics kill viruses	True	10	4.2
	False	225	93.4
	Unknown	6	2.5
Overuse of antibiotics makes them become ineffective	True	228	94.6
	False	8	3.3
	Unknown	5	2.1
Bacteria can become resistant to antibiotics	True	241	100
	False	0	0
	Unknown	0	0
Healthy people and animals can carry antibiotic resistant bacteria	Agree	190	78.8
	Neither agree/nor disagree	26	10.8
In what microbes is antibiotic resistance a major problem?*	Bacteria	236	97.9
	Fungi	53	22.0
	Protozoa	48	19.9
	Helminths	38	15.8
	Viruses	30	12.5
Do you think overuse of antibiotics can lead to development of resistant organisms?	No	6	2.5
	Yes	227	94.2
	Don't know	8	3.3
A withdrawal period does not have to be observed for milking cows treated with antibiotics such as penicillin before milk can be consumed	True	14	5.8
	False	225	93.4
	Unknown	2	0.8
A withdrawal period has to be strictly observed in treated poultry before any poultry product is passed as fit for human consumption	True	236	97.9
	False	4	1.7
	Unknown	1	0.4
What is the difference between an antimicrobial and an antibiotic?	Antibiotics kill and or stop the growth of bacteria, whereas antimicrobials kill viruses, fungi or bacteria	197	81.8
	Antibiotics are naturally occurring compounds and not synthetic compounds	24	10
	An antibiotic is the same as an antimicrobial	16	6.7
	Don't know	4	1.7

Have you ever heard the term "antibiotic stewardship" or "antimicrobial stewardship"?	No	152	63.1
	Yes	89	36.9
Which of the following do you think best defines antibiotic stewardship or antimicrobial stewardship? **	All of the above	61	68.5
	A coordinated intervention designed to improve and measure the appropriate use of antibiotics by promoting the selection of the optimal antimicrobial drug regimen, dose, duration of therapy and route of administration.	11	12.4
	Optimal selection, dosage, and duration of antibiotic treatment that results in the best clinical outcome for the treatment or prevention of infection, with minimal toxicity to the patient and minimal impact on subsequent resistance	9	10.1
	A systematic effort to educate and persuade prescribers of antibiotics to follow evidence-based prescribing, in order to stem antibiotic overuse, and thus antibiotic resistance.	8	9
	None of the above	0	0
Have you read or heard of the Nigeria Center for Disease Control 5-point action plan for responsible use of antimicrobials?	No	144	59.8
	Yes	97	40.3
Of the following, which topics would you like to receive more information on?*	Links between the health of humans, animals and the environment	182	75.5
	Guidelines to use of antibiotics	148	61.4
	Resistance to antibiotics and how resistance develops	146	60.6
	Critically important antimicrobials	144	59.8
	Animal disease conditions for which antibiotics are used or should be used	143	59.3
	Microbiological culture and sensitivity testing	134	55.6

* The results for this variable are presented as row percentages instead of column percentages, as such the column percentages for this variable do not add up to 100%.
**The denominator used for this question was 89. Only respondents that answered “yes” (n=89) to the previous question “Have you ever heard the term "antibiotic stewardship" or "antimicrobial stewardship"?” were assessed in this question.

Table S2. Attitudes of respondents towards antibiotic use and antimicrobial resistance (AMR)

Variables	Response	Frequency (n=241)	Percentage (%)
Of the following potential contributors to antibiotic resistance, which ones do you consider the most important?*	Excessive use of antibiotics in livestock and food production	202	83.8
	Under-dosing of antibiotics	185	76.8
	Long duration of antibiotic treatments	132	54.8
	Public awareness of antibiotic resistance	130	53.9
	Too many broad-spectrum antibiotics used	123	51.0
	Poor biosecurity practices	68	28.2
In your opinion, which of the following are important challenges in the world today?*	Antibiotic resistance	195	80.9
	Food security	180	74.7
	Climate change	146	60.9
	Gender inequality	40	16.6
	Obesity	19	7.9
When there is poor biosecurity, prophylactic antibiotics are an appropriate alternative to protect animal health	Disagree	88	36.5
	Agree	112	46.5
	Neither agree/nor disagree	41	17.0
Biosecurity is important in food production	Disagree	2	0.8
	Agree	237	98.3
	Neither agree/nor disagree	2	0.8
Do veterinarians have a role to play in preventing public health threats posed by antibiotic resistance?	No	0	0
	Yes	240	99.6
	Unknown	1	0.4
Do you believe that prescribing unnecessary antibiotics is professionally unethical?	No	6	2.5
	Yes	234	97.1
	Unknown	1	0.4
Do you think that antibiotic resistance is a national problem?	No	5	2.1
	Yes	233	96.7
	Unknown	3	1.2
Do you think we have enough antibiotics under development at the moment to keep up with the problem of resistance?	No	145	60.2
	Yes	59	24.5
	Unknown	37	15.4
Do you think that antibiotic resistance will be a greater problem later in your career than it is today?	No	9	3.7
	Yes	223	92.5
	Unknown	9	3.7
	No	48	19.9

Do you believe that the antibiotics you prescribe may contribute to the problem of antibiotic resistance?	Yes	172	71.4
	Unknown	21	8.7
Do you feel you have sufficient knowledge on antibiotic use?	No	102	42.3
	Yes	117	48.5
	Unknown	22	9.1

* The results for this variable are presented as row percentages instead of column percentages, as such the column percentages for this variable do not add up to 100%.

Table S3. Contributors to antibiotic resistance, as reported by respondents

**Of the following potential contributors to antibiotic resistance, which ones do you consider the most important? ***	**Frequency (n=241)**	**Percentage (%)**
Excessive use of antibiotics in livestock and food production	202	83.8
Under-dosing of antibiotics	185	76.8
Long duration of antibiotic treatments	132	54.8
Public awareness of antibiotic resistance	130	53.9
Too many broad-spectrum antibiotics used	123	51.0
Poor biosecurity practices	68	28.2

* The results for this variable are presented as row percentages instead of column percentages, as such the column percentages for this variable do not add up to 100%.

Table S4. Factors influencing antibiotic prescription practices and choice

Variables	Response	Frequency (n=241)	Percentage (%)
Does your practice have a standardized protocol for treating sick animals with antibiotics?	No	71	29.5
	Yes	154	63.9
	Don't know	16	6.6
How often do you encounter owner-initiated antibiotic treatment before presentation?	Never	13	5.4
	Sometimes (1-3 times a month)	75	31.1
	Frequently (> 3 times a month)	132	54.8
	Don't know	21	8.7
When a patient presents for the first time and antibiotics are indicated, what guidelines do you follow to help you select the appropriate antibiotic?*	Empirical treatment whilst awaiting AST** results	144	59.8
	Microbiological culture and AST	128	53.1
	Use whatever antibiotic is available	45	18.7
	Use whatever antibiotic the patient can afford	35	14.5
	Don't know	7	2.9
Which of the following guidelines influence your decision the most when selecting an antibiotic to use?*	Veterinary education/training	191	79.3
	Prescription guidelines/policies supplied by vet hospital/bodies/entities	166	68.9
	Product label/leaflet (manufacturer recommendations)	155	64.3
	Legal restriction of drug to a defined species	93	38.6
	Published literature	85	35.3
Which of the following factors influence your decision the most important when selecting an antibiotic to use?*	Owner ability to pay	196	81.3
	Cost of antibiotic drug	195	80.9
	Profit margin to vet	66	27.4
	Marketing offers	40	16.6
	Advertisement by drug representative	31	12.9
	Drug sellers	20	8.3
Which of the following past experiences influence your decision the most important when selecting an antibiotic to use?*	Vet previous experience	232	96.3
	Advice from colleagues	164	68.0
	Owner preference for a specific antibiotic	23	9.5
Of the following antibiotic characteristics, which ones influence your decision the most important when selecting an antibiotic to use?*	Spectrum of action of antibiotic	206	85.5
	Sensitivity results/antibiogram	152	63.1
	Withdrawal period	122	50.6
	Ease of administration	107	44.4
	Risk of development of antibiotic resistance	104	43.2

* The results for this variable are presented as row percentages instead of column percentages, as such the column percentages for this variable do not add up to 100%.

** AST: antimicrobial susceptibility testing.

Table S5. Frequency of antimicrobial susceptibility testing (AST) use and barriers to AST use

Variables	Response	Frequency (n=241)	Percentage (%)
How often do you undertake antimicrobial sensitivity testing before starting antibiotic treatment?	Never	69	28.6
	Sometimes (1-3 times/month)	112	46.5
	Frequently (> 3 times/month)	49	20.3
	Don't know	11	4.6
Which of the following influence your decision the most when requesting AST?*	Poor response to initial antimicrobial therapy or therapeutic failure	182	75.5
	Recurrent conditions	170	70.5
	No knowledge of farm or animal health status	64	26.6
	Owner request	44	18.3
Of the following barriers to using AST, which barriers do you consider the most important?*	Unavailability of lab services to conduct microbiological culture and sensitivity testing	198	82.2
	Owner unable or not willing to pay for culture and sensitivity testing	173	71.8
	Case is urgent, requires immediate antibiotic therapy	137	56.8
	Long waiting time for sensitivity results	85	35.3
	Not sure of what to request from the lab	8	3.3

* The results for this variable are presented as row percentages instead of column percentages, as such the column percentages for this variable do not add up to 100%.

Table S6. Practices and factors influencing respondents' decisions on selecting antibiotics and antimicrobial susceptibility testing (AST) use

Variables	Response	Frequency (n=241)	Percentage (%)
Does your practice have a standardised protocol for treating sick animals with antibiotics?	No	71	29.5
	Yes	154	63.9
	Don't know	16	6.6
How often do you encounter owner-initiated antibiotic treatment before presentation?	Never	13	5.4
	Sometimes (1-3 times a month)	75	31.1
	Frequently (> 3 times a month)	132	54.8
	Don't know	21	8.7
When a patient presents for the first time and antibiotics are indicated, what guidelines do you follow to help you select the appropriate antibiotic?*	Empirical treatment whilst awaiting MCS result	144	59.8
	Microbiological culture and sensitivity (MCS) testing	128	53.1
	Use whatever antibiotic is available	45	18.7
	Use whatever antibiotic the patient can afford	35	14.5
	Don't know	7	2.9
How often do you undertake antimicrobial sensitivity testing before starting antibiotic treatment?	Never	69	28.6
	Sometimes (1-3 times a month)	112	46.5
	Frequently (more than 3 times a month)	49	20.3
	Don't know	11	4.6
Which of the following influence your decision the most important when requesting antimicrobial sensitivity testing?*	Poor response to initial antimicrobial therapy or therapeutic failure	182	75.5
	Recurrent conditions	170	70.5
	No knowledge of farm or animal health status	64	26.6
	Owner request	44	18.3
Of the following barriers to using antimicrobial sensitivity testing, which barriers do you consider the most important?*	Unavailability of lab services to conduct microbiological culture and sensitivity testing	198	82.2
	Owner unable or not willing to pay for culture and sensitivity testing	173	71.8
	Case is urgent, requires immediate antibiotic therapy	137	56.8
	Long waiting time for sensitivity results	85	35.3
	Not sure of what to request from the lab	8	3.3
Which of the following guidelines influence your decision the most when selecting an antibiotic to use?*	Veterinary education/training	191	79.3
	Prescription guidelines/policies supplied by vet hospital/bodies/entities	166	68.9
	Product label/leaflet (manufacturer recommendations)	155	64.3
	Legal restriction of drug to a defined species	93	38.6
	Published literature	85	35.3
Which of the following factors influence your	Owner ability to pay	196	81.3
	Cost of antibiotic drug	195	80.9

decision the most important when selecting an antibiotic to use?*	Profit margin to vet	66	27.4
	Marketing offers	40	16.6
	Advertisement by drug representative	31	12.9
	Drug sellers	20	8.3
Which of the following past experiences influence your decision the most important when selecting an antibiotic to use?*	Vet previous experience	232	96.3
	Advice from colleagues	164	68.0
	Owner preference for a specific antibiotic	23	9.5
Of the following antibiotic characteristics, which ones influence your decision the most important when selecting an antibiotic to use?*	Spectrum of action of antibiotic	206	85.5
	Sensitivity results/antibiogram	152	63.1
	Withdrawal period	122	50.6
	Ease of administration	107	44.4
	Risk of development of antibiotic resistance	104	43.2

* The results for this variable are presented as row percentages instead of column percentages, as such the column percentages for this variable do not add up to 100%.

Table S7. Relationship between the use of antimicrobial susceptibility testing (AST) before antibiotic treatment and select investigated variables.

Variable	Categories	Did not use AST before antibiotics treatment (%) n=69	Used AST before antibiotics treatment (%) n=161	*P* value
Age group	25-34 years old	39 (56.5)	72 (44.7)	0.207
	35-44 years old	22 (31.9)	59 (36.6)	
	45 years old or more	8 (11.6)	30 (18.6)	
Gender	Male	54 (78.3)	129 (80.1)	0.748
	Female	15 (21.7)	32 (19.9)	
Educational level	Veterinary degree	44 (63.8)	99 (61.5)	0.744
	Additional education	25 (36.2)	62 (38.5)	
Years in practice	0-<5 years	34 (49.3)	48 (29.8)	0.049
	5-10 years	17 (24.6)	56 (34.8)	
	10-15 years	8 (11.6)	21 (13)	
	16 years and above	10 (14.5)	34 (21.1)	
	Prefer not to say	0 (0)	2 (1.2)	
Type of practice*	Mixed practice	51 (73.9)	96 (59.6)	0.247
	Large animal practice	2 (2.9)	13 (8.1)	
	Poultry practice	4 (5.8)	18 (11.2)	
	Small animal practice	6 (8.7)	19 (11.8)	
	Other practice type	2 (2.9)	8 (5)	
	Do not practice	4 (5.8)	7 (4.3)	
Practice location*	South-West	9 (13)	29 (18)	0.369
	North-West	17 (24.6)	29 (18)	
	South-South	2 (2.9)	14 (8.7)	
	North-East	17 (24.6)	28 (17.4)	
	North-Central	18 (26.1)	46 (28.6)	
	South-East	6 (8.7)	15 (9.3)	
Knowledge of antimicrobial stewardship (AMS)*	Never heard of AMS	50 (72.5)	95 (59)	0.032
	All of three correct definitions of AMS selected	16 (23.2)	41 (25.5)	
	At least one of three correct definitions of AMS selected	3 (4.3)	25 (15.5)	
Knowledge of NCDC 5 points	No	51 (73.9)	85 (52.8)	0.003
	Yes	18 (26.1)	76 (47.2)	
Prophylactic use of antibiotics is appropriate when biosecurity is poor	Neither agree/nor disagree	17 (24.6)	20 (12.4)	0.029
	Agree	33 (47.8)	74 (46)	
	Disagree	19 (27.5)	67 (41.6)	
Owner initiated treatment	Never	6 (8.7)	6 (3.7)	0.059
	Frequently	38 (55.1)	91 (56.5)	
	Sometimes	14 (20.3)	58 (36)	
	Do not know	11 (15.9)	6 (3.7)	
AB treatment protocol in practice	No	31 (44.9)	34 (21.1)	<0.001
	Yes	32 (46.4)	118 (73.3)	
	Do not know	6 (8.7)	9 (5.6)	

*Fisher's exact test used. NCDC: Nigeria Center for Disease Control

Table S8. Relationship between knowledge level on appropriate antibiotic (AB) use and antimicrobial resistance (AMR) and select investigated variables.

Variable	Categories	Low-moderate knowledge (%) n=42	High knowledge (%) n=198	P value
Age group*	25-34 years old	17 (40.5)	99 (50)	0.024
	35-44 years old	22 (52.4)	63 (70.1)	
	45 years old or more	3 (7.1)	36 (18.2)	
Gender	Male	35 (83.3)	156 (78.8)	0.507
	Female	7 (16.7)	42 (21.2)	
Educational level	Veterinary degree	33 (78.6)	119 (60.1)	0.024
	Additional education/training	9 (21.4)	79 (39.9)	
Years in practice	0-<5 years	19 (45.2)	68 (34.3)	0.304
	5-10 years	11 (26.2)	64 (32.3	
	10-15 years	7 (16.7)	23 (11.6)	
	16 years and above	5 (11.9)	41 (20.7)	
	Prefer not to say	0 (0)	2 (1)	
Type of practice*	Mixed practice	28 (66.7)	125 (63.1)	0.806
	Large animal practice	4 (9.5)	13 (6.6)	
	Poultry practice	3 (7.1)	20 (10.1)	
	Small animal practice	3 (7.1)	22 (11.1)	
	Other practice type	1 (2.4)	9 (4.5)	
	Do not practice	3 (7.1)	9 (4.5)	
Employment type	Private practice	19 (45.2)	73 (36.9)	0.659
	Government employee	15 (35.7)	60 (30.3)	
	Non-governmental organisation employee	4 (9.5)	18 (9.1)	
	Teaching	3 (7.1)	22 (11.1)	
	Research	1 (2.4)	14 (7.1)	
	Other	0 (0)	11 (5.6)	
Practice location*	South-West	9 (21.4)	30 (15.2)	0.872
	North-West	9 (21.4)	40 (20.2)	
	South-South	3 (7.1)	14 (7.1)	
	North-East	8 (19)	38 (19.2)	
	North-Central	11 (26.2)	57 (28.8)	
	South-East	2 (4.8)	19 (9.6)	
Heard of antimicrobial stewardship before?	No	28 (66.7)	123 (62.1)	0.58
	Yes	14 (33.3)	75 (37.9)	
Knowledge of NCDC 5 points	No	25 (59.5)	118 (59.6)	0.993
	Yes	17 (40.5)	80 (40.4)	
Prophylactic use of antibiotics is appropriate when biosecurity is poor*	Neither agree/nor disagree	13 (31)	28 (14.1)	<0.001
	Agree	27 (64.3)	84 (42.4)	
	Disagree	2 (4.8)	86 (43.4)	
AB treatment protocol in practice	No	11 (26.2)	60 (30.3)	0.62
	Yes	28 (66.7)	126 (63.6)	
	Do not know	3 (7.1)	12 (6.1)	

Use of AST before AB treatment	No	11 (12.1)	58 (28.3)	0.704
	Yes	29 (69)	132 (66.7)	
	Do not know	2 (4.8)	8 (4)	

*Fisher's exact test used. NCDC: Nigeria Center for Disease Control. AST: antimicrobial susceptibility testing.

Table S9. Scoring of knowledge level on appropriate antibiotic (AB) use and antimicrobial resistance (AMR). For each question, a score was assigned based on the response with 0 given for an incorrect response and 1 for a correct response.

**Variables**	**Response**	**Frequency (n=241)**	**Percentage (%)**	**Score**	**Total max score**
Antibiotics kill or stop the growth of both good and bad bacteria	True	220	91.3	1	1
	False	20	8.3	0	
	Unknown	1	0.4	0	
Antibiotics kill viruses	True	10	4.2	0	1
	False	225	93.4	1	
	Unknown	6	2.5	0	
Bacteria can become resistant to antibiotics	True	241	100	1	1
	False	0.0	0.0	0	
	Unknown	0.0	0.0	0	
Healthy people and animals can carry antibiotic resistant bacteria	Agree	190	78.8	1	1
	Neither agree/nor disagree	26	10.8	0	
	Disagree	0	0.0	0	
In what microbes is antibiotic resistance a major problem?	Bacteria alone	150	62.24	1	1
	Bacteria and/or other pathogens (Fungi, viruses)	91	37.75	0	
Do you think overuse of antibiotics can lead to development of resistant organisms?	No	6	2.5	0	1
	Yes	227	94.2	1	
	Don't know	8	3.3	0	
A withdrawal period does not have to be observed for milking cows treated with antibiotics before milk can be consumed	True	14	5.8	0	1
	False	225	93.4	1	
	Unknown	2	0.8	0	
A withdrawal period must be strictly observed in treated poultry before any poultry product is passed as fit for human consumption	True	236	97.9	1	1
	False	4	1.7	0	
	Unknown	1	0.4	0	
When there is poor biosecurity, prophylactic antibiotics are an appropriate alternative to protect animal health	Disagree	88	36.5	1	1
	Agree	112	46.5	0	
	Neither agree/nor disagree	41	17.0	0	
